# *NUDT15* and *TPMT* polymorphisms in three distinct native populations of the Brazilian Amazon

**DOI:** 10.3389/fphar.2024.1359570

**Published:** 2024-02-06

**Authors:** Jamila Alessandra Perini, Paulo Cesar Basta, Guilherme Suarez-Kurtz

**Affiliations:** ^1^ Laboratório de Pesquisa de Ciências Farmacêuticas (LAPESF), Universidade do Estado do Rio de Janeiro (UERJ), Rio de Janeiro, Brazil; ^2^ Departamento de Endemias Samuel Pessoa, Escola Nacional de Saúde Pública, Fundação Oswaldo Cruz (ENSP/Fiocruz), Rio de Janeiro, Brazil; ^3^ Divisão de Pesquisa Clínica e Desenvolvimento Tecnológico, Coordenação de Pesquisa, Instituto Nacional de Câncer, Rio de Janeiro, Brazil

**Keywords:** Brazilian Amazon, native ancestry, population diversity, genetic polymorphism, NUDT15, TPMT, thiopurine drug, pharmacogenetics

## Abstract

This is the first report of the distribution of *TPMT* and *NUDT15* single nucleotide polymorphisms and metabolic phenotypes associated with cytotoxicity of thiopurine drugs, in indigenous groups of Brazilian Amazon: Munduruku, Paiter-Suruí and Yanomami. The minor allele frequency (MAF) of *NUDT15* rs116855232 did not differ significantly across the groups; *TPMT* rs1800462 was absent, while rs1800460 and rs1142345 were in strong linkage disequilibrium, and 10- and 30-fold more common in Paiter-Suruí. Indeed, the MAFs in Paiter-Surui (0.193 and 0.188) are the largest report globally. The distribution of combined NUDT15/TPMT metabolic phenotypes differed significantly (*p* < 0.0001) and largely (Cramér´s V = 0.37) across cohorts. This has important pharmacogenetic implications: the Clinical Pharmacogenetics Implementation Consortium recommendations to reduce or consider reduction of thiopurine dose applies to 4.4% Yanomami, 5.6% Munduruku, *versus* 41% Paiter-Suruí. The proportion of Paiter-Suruí at risk of thiopurine intolerance is 3- to 4-fold higher than any other population worldwide.

## 1 Introduction

Population diversity impacts pharmacogenetics/pharmacogenomics (PGx) research and clinical implementation. This is most relevant to Native populations of the Americas, considering that “the Americas, especially South America, show extreme genetic variability” (Cavalli-Sforza et al., 1994) and that PGx studies of Native American populations are scarce. Although most common PGx markers are shared across global populations (Jakobsson et al., 2008), there are large differences in frequency of key PGx variants which have major influence on drug response (Zhou et al., 2017). A remarkable example is the *NUDT15* c.415C>T (rs116855232) single nucleotide polymorphism (SNP), linked to cytotoxicity of thiopurine drugs, widely used for chemotherapy of acute lymphoblastic leukemia (ALL) and inflammatory bowel disease (Relling et al., 2019). The minor c.415T allele of rs116855232, associated with a dramatic loss of NUDT15 function and increased risk of thiopurine cytotoxicity, is relatively common in East Asians, rare or absent in Europeans and Africans, and shows the highest frequency worldwide (0.25–0.31) in Kaingang Amerindians from Brazil (Suarez-Kurtz et al., 2020). However, in another Native group from Brazil, namely Xavante, the minor allele frequency (MAF) of rs116855232 is considerably lower (0.05) (Suarez-Kurtz et al., 2020), an example of the rich genetic diversity of Native American peoples (Salzano and Bortolini, 2002).

The risk of thiopurine cytotoxicity is also linked to polymorphisms in another pharmacogene, namely *TPMT*, which encodes the thiopurine metabolizing enzyme, TPMT. Accordingly, the Clinical Pharmacogenetic Implementation Consortium (CPIC) and the Dutch Pharmacogenetics Working Group (DPWG) guidelines for thiopurines are based on TPMT, NUDT15 and compound (combined TPMT and NUDT15) metabolic phenotypes, inferred from the individual TPMT and NUDT15 diplotypes (Relling et al., 2019; https://www.pharmgkb.org/guidelineAnnotation/PA166104952). To our knowledge, there is only one published study of the frequency of both *NUDT15* and *TPMT* polymorphisms and predicted phenotypes in Native peoples of the Americas (Amerindians): Ferreira and others (2020), enrolled self-reported Natives from the Brazilian Amazon, but cautioned that “no study participant was living in Indigenous reservation areas, which may account for considerable admixture of Native, European and to a lesser extent, African ancestry” in the study cohort. Indeed, extensive admixture of these three major ancestral roots is predominant in the present-day Brazilian population, irrespective of self-reported “race/Color” (Pena et al., 2011). Notably, self-reported Indigenous (the term used in the Brazilian Census to designate Natives/Amerindians) amounted to 1,652,876 people, which is less than 1.0% of the entire population (https://censo2022.ibge.gov.br/). Despite the small number, indigenous people set up a tremendous sociocultural diversity, they represent more than 300 ethnic groups, speaking almost 300 languages (Bastos et al., 2017).

The present study investigated the distribution of *NUDT15* and *TPMT* polymorphisms listed in CPIC and DPWG thiopurine guidelines, and genotype-predicted metabolic phenotypes in three major indigenous groups, namely Munduruku, Paiter-Suruí and Yanomami, living in reservation areas in the Brazilian Amazon (Table 1). Currently, the Munduruku population numbers around 14,000 people, spread over approximately 150 villages and is mainly concentrated in three Indigenous Lands (Munduruku, Sai Cinza, and Sawré Muybu) in the states of Pará (municipalities of Santarém, Itaituba, Jacareacanga). The Paiter-Surui population numbers approximately 1,500 people living in 22 villages distributed across the Sete de Setembro Indigenous Land on the border of the states of Rondônia and Mato Grosso. The Yanomami number approximately 29,000 inhabitants distributed in 365 villages in the Yanomami Indigenous Land in the state of Roraima and Amazonas. The three populations live in the northern region of Brazil (Supplementary Figure S1).

**TABLE 1:** Characteristics of three native distinct groups living in reservation areas in Brazil.

	Munduruku (n = 90)	Paiter-Suruí (n = 90)	Yanomami (n = 90)
Localities (river basin)	Tapajós	Madeira	Branco
Geographical coordinates^a^			
Y	4° 44′ 35" (S)	11° 13′ 16" (S)	2° 45′ 29" (N)
X	56° 24′ 2" (W)	61° 17′ 56" (W)	62° 13′ 22" (W)
Region	North (Pará)	North (Rondônia)	North (Roraima)
Linguistic group	Tupi	Tupi-Mondé	Yanoman/Ninam
Villages	Sawré Muybu, Poxo Muybu and Sawré Aboy	Lapetanha, Gamir, Riozinho, Pabekepi, Joaquim, Tikã	Castanha, Pewaú, Cajú, Ilha, Ilhimakok, Lasasi, Milikowai, Porapi, Uxiu

^a^ Geographic coordinates of the villages where recruitment took place: Sawré Muybu (Munduruku), Lapetanha (Paiter-Suruí) and Lasasi Yanomami).

## 2 Materials and methods

This study is within the scope of a project described by Basta and others (2021) approved by the Brazilian National Ethics Committee of Human Research (protocol number 65671517.1.0000.5240). The study participants were recruited in three distinct indigenous reservation areas in the Brazilian Amazon: Munduruku (from Sawré Muybu Indigenous land), Paiter-Suruí (from Sete de Setembro Indigenous land) and Yanomami (from the Ninam subgroup) (Supplementary Figure S1). From each group, 90 adults not related by blood were selected, adding up 270 participants in the overall study cohort. Additional information on Munduruku, Paiter-Suruí and Yanomami groups is presented in Supplementary Figure S1. All participants provided written informed consent, before enrollment, and the leaders of the groups were apprised, and granted approval for publication, of the results of the study.

Samples from oral mucosa epithelial cells collected using sterile swabs were stored in a buffered solution, individually identified, and transported to the Laboratory of Pharmaceutical Science (https://lapesfuerjzo.my.canva.site/#in%C3%ADcio) of the State University of Rio de Janeiro, in Rio de Janeiro-RJ, Brazil. Genomic DNA was extracted following the procedures recommended by the manufacturer using a QIAamp DNA mini kit (catalog number 51304, Qiagen, Hilden, Germany), as previously described (Silva et al., 2023). Validated Taqman assays (Applied Biosystems, Foster City, CA, United States of America) were employed for allele discrimination of *NUDT15* (C_154823200_10) rs116855232 (c.415C>T), *TPMT* (C_12091552_30) rs1800462 (c.238G>C), (C_30634116_20) rs1800460 (c.460G>A) and (C_19567_20) rs1142345 (c.719A>G). TPMT*2, *3A, *3B and *3C haplotypes were defined according to the TPMT Definition Table; TPMT, NUDT15 and compound metabolic phenotypes were assigned according to the Allele Functionality Tables in the CPIC thiopurine guideline (Relling et al., 2019).

Chi square tests, available at https://www.icalcu.com/stat/chisqtest.html were applied to assess deviations of genotype distribution from Hardy-Weinberg equilibrium (HWE) and to compare the distribution of alleles, genotypes and predicted metabolic phenotypes across the study cohorts. The Cramér´s V test (Cramér, 1946), an effect size measurement for chi-square tests, was applied to assess the strength (magnitude) of statistically significant associations observed with the chi square tests. Cramér’s V (range 0–1) is calculated as V = √(X2/n*df), where X2: is the chi-square statistic, n is the total sample number, df is the degree of freedom estimated as min (c-1, r-1), in which c: number of columns and r: number or rows. The Cramér’s V values are interpreted according to the Cohen´s h scale (Cohen, 1988), which designates the effect size as negligible, small, intermediate and large. The LDpair Tool (https://ldlink.nih.gov/?tab=ldpair) was used to assess linkage disequilibrium (LD) between TPMT SNPs in the One Thousand Genomes Project (1KGP) superpopulations.

The FST statistics (Wright, 1978) was applied to characterize genetic differentiation (divergence) at the interrogated loci in *NUDT15* and *TPMT* among the indigenous groups. The allele-specific FST values are calculated as: FST = HT–HS, where HS and HT refer, respectively, to heterozygosity across subpopulations of a group and heterozygosity within the overall group. FST values range from 0 (indicating no genetic divergence) to 1 (indicating fixation for alternative alleles in different subpopulations). The qualitative guidelines proposed by Wright (1978) for interpretation of FST were adopted: FST values < 0.05 indicate little genetic differentiation; 0.05–0.15, moderate genetic differentiation; 0.15–0.25, large genetic differentiation and FST >0.25, very large differentiation.

## 3 Results

Table 2 shows the minor allele frequency (MAF) and genotype distribution of the interrogated *NUDT15* and *TPMT* SNPs in the three indigenous groups; no deviation from HWE was observed. The MAF of *NUDT15* rs116855232 ranged from 0.017 in Yanomami to 0.072 in Paiter-Suruí, with an FST value (0.02) associated with small genetic divergence. The distribution of *NUDT15* genotypes did not differ significantly across the three groups.

**TABLE 2:** Allele frequency and genotype distribution of *NUDT15* and *TPMT* polymorphisms.

Polymorphisms	Alleles	Yanomami	Munduruku	Paiter-Suruí	*F*_*st*_ test	Genotypes	Yanomami	Munduruku	Paiter-Suruí	*p*-Value
*NUDT15* rs116855232	C	0.983	0.973	0.928	0.02	C/C	0.967	0.957	0.878	0.12
	T	0.017	0.027	0.072		C/T	0.033	0.033	0.100	
						T/T	0.0	0.011	0.022	
*TPMT* rs1142345	A	0.994	0.984	0.807	0.11	A/A	0.989	0.967	0.648	0.0001
	G	0.006	0.016	0.193		A/G	0.011	0.033	0.318	
						G/G	0.0	0.0	0.034	
*TPMT* rs1800460	G	0.994	0.984	0.813	0.11	G/G	0.989	0.967	0.648	0.0001
	A	0.006	0.016	0.188		G/A	0.011	0.033	0.330	
						A/A	0.0	0.0	0.023	
*TPMT* rs1800462	G	1.0	1.0	1.0	n/a	G/G	1.0	1.0	1.0	n/a
	A	0.0	0.0	0.0		G/A	0.0	0.0	0.0	
						A/A	0.0	0.0	0.0	

*F*_*ST*_, test was applied to assess genetic differentiation: <0.05 little genetic differentiation; 0.05–0.15 moderate; 0.15–0.25 large; and >0.25 very large. *p*-value from Chi-square test. n/a = not determined.

Regarding the *TPMT* SNPs, rs1800462 was absent in the overall cohort, whereas rs1142345 and rs1800460 were present at 10- to 30-fold higher MAF in Paiter-Suruí (0.193 and 0.188, respectively) than in Munduruku (0.016, both SNPs) or Yanomami (0.006, both SNPs). The FST statistics showed moderate genetic divergence for both rs1142345 and rs1800460 SNPs, the chi-square test indicated highly significant differences in the distribution of rs1142345 and rs1800460 genotypes and the Cramér V test pointed to large magnitude of effects on the distribution of genotypes (Table 2). The rs1142345 and rs1800460 were in nearly perfect LD in the overall cohort (D´ = 0.96; R2 = 0.86); this finding combined with the absence of the rs1800462 SNP (which defines the TPMT*2 allele) resulted in TPMT*3A being the only (Munduruku and Yanomami) or the major (Paiter-Suruí) variant *TPMT* star allele identified in the participants; TMPT*3B (rs1800460 only) and *3C (rs1142345 only) were detected in two and one Paiter-Suruí, respectively (Table 3). The distribution of the *TPMT* star alleles differed significantly across the three groups (*p* < 0.0001) and the Cramér´s V test indicated large magnitude of effect.

**TABLE 3:** Frequency of *TPMT* star alleles.

*TPMT* alleles	Yanomami	Munduruku	Paiter-Suruí	Statistical analysis
*1	0.994	0.984	0.803	*p* < 0.0001
*3A	0.006	0.016	0.180	
*3B	0.0	0.0	0.011	V = 0.23
*3C	0.0	0.0	0.006	
				

*p*-value from Chi-square test. V test was applied to assess the strength of statistically significant associations.

The frequency distribution of diplotype-inferred NUDT15, TPMT and compound metabolic phenotypes in Munduruku, Paiter-Suruí and Yanomami is shown in Supplementary Table S1. No significant difference was observed in relation to NUDT15: the normal metabolic phenotype (NM) predominated largely in all groups (frequency 0.88–0.97), while the frequency of IMs and PMs ranged between 0.03–0.10 and zero to 0.02, respectively. By contrast, the distribution of the TPMT phenotypes differed markedly between Paiter-Suruí and the other two groups: although the NM phenotype was predominant in all groups, its frequency in Paiter-Suruí (0.64) was considerably lower than in Yanomami (0.99) or Munduruku (0.97); IMs ranged in frequency between 0.01 and 0.03 in the latter two groups *versus* 0.33 in Paiter-Suruí, while the PM phenotype was not detected in Munduruku and Yanomami, but was assigned to three Paiter-Suruí (frequency 0.03). The Cramér´s V statistics indicated large magnitude of effects on the distribution of TMPT phenotypes across the groups (V = 0.33).

**FIGURE 1:**
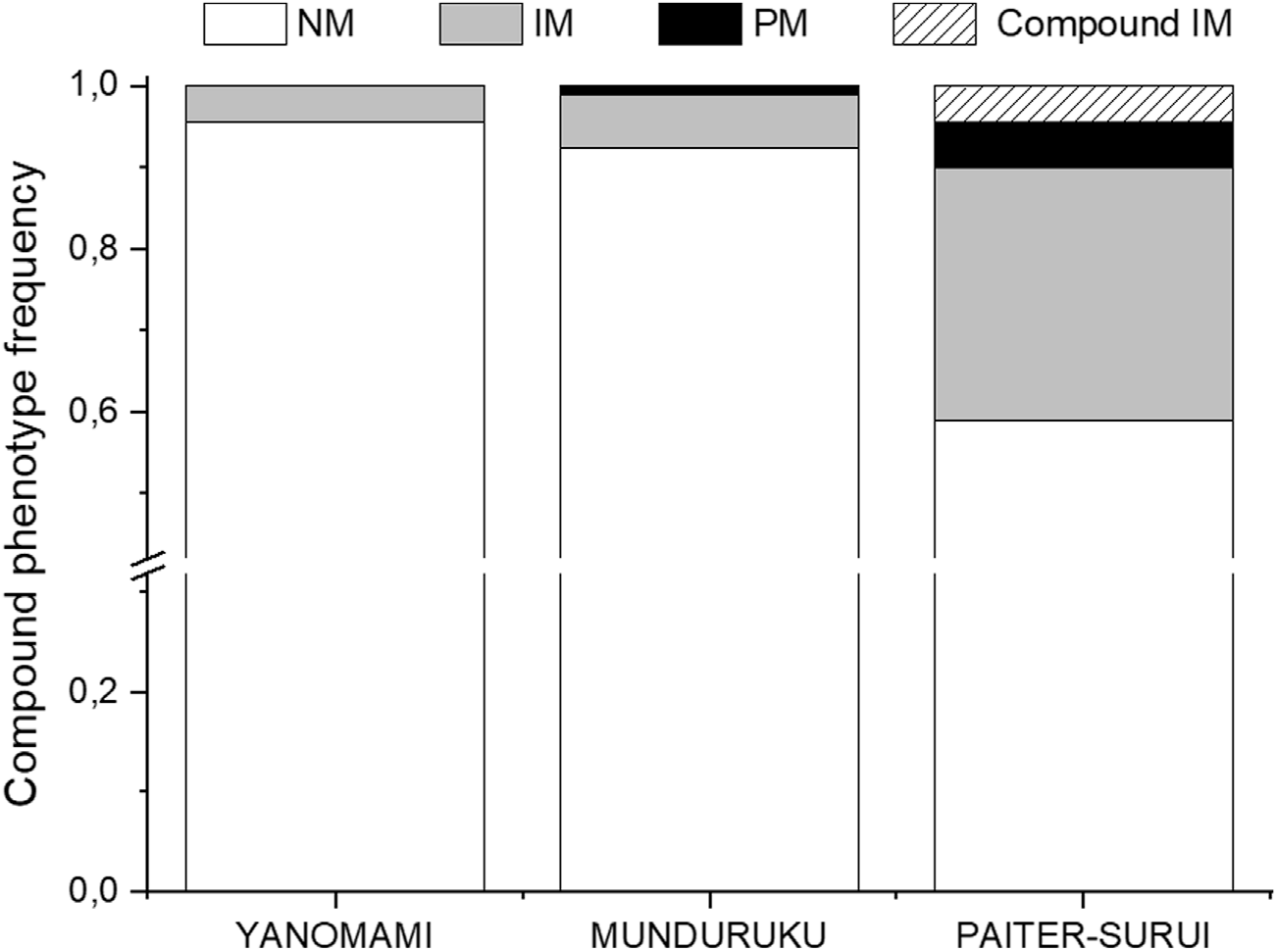
Distribution of the compound NUDT15 and TMPT metabolic phenotypes across the study cohorts. NM, normal metabolizer for both enzymes; IM, intermediate metabolizer for either enzyme; PM, poor metabolizer for one or both enzymes; Compound IM, intermediate metabolizer for both enzymes. Data from 90 individuals in each group (Munduruku, Paiter-Suruí and Yanomami).

The distribution of the compound NUDT15/TPMT phenotypes differed significantly (*p* < 0.001) and largely (Cramér´s V = 037) across the three groups (Supplementary Table S1 and Figure 1). Of notice, the compound IM phenotype, i.e. IMs for both NUDT15 and TPMT, were present only in Paiter-Suruí (4 individuals). According to the compound NUDT15/TPMT phenotypes, the CPIC guideline recommendations for thiopurine dosing: (1) use the standard initial dose, applies to 95.6% Yanomami, 92.4% Munduruku and 58.9% Paiter-Suruí; 2) consider initial dose reduction in IMs of either gene, applies to 4.4% Yanomami, 6.5% Munduruku and 31.1% Paiter-Suruí; (3) reduce the initial dose in PMs of NUDT15 and/or TPMT, and in IMs of both genes, applies to none Yanomami, 1.1% Munduruku and 10.0% Paiter-Suruí.

## 4 Discussion

This is the first study of the distribution of both *NUDT15* and *TPMT* clinically relevant polymorphisms and respective metabolic phenotypes in Native Americans living in indigenous reservation areas. Of the three groups included in this study, only Paiter-Suruí have been previously genotyped for *NUDT15* and *TMTP* polymorphisms, in the context of the Human Genome Diversity Project (HGDP) (Cavalli-Sforza, 2005). The small number of Paiter-Suruí samples in the HGDP (n = 8) might account for the discrepancy with our findings regarding *NUDT15* rs116855232, which was not detected in the HGDP samples (https://cephb.fr/en/hgdp/hgdp_search.php), but showed a MAF of 0.072 in this study (n = 90). Regarding *TPMT* SNPs, rs1800462 was absent, while rs1800460 and rs1142345 were common in both cohorts, with MAFs of 0.125 (both SNPs) in HGDP and 0.188–0.193 in this study.

The MAFs of rs1800460 and rs1142345 in the Paiter-Suruí enrolled in our study were several fold higher than in Yanomami (0.006, both SNPs) or Munduruku (0.016, both SNPs), and exceeded largely the MAFs reported for multiethnic Native cohorts from the Brazilian Amazon (0.043 and 0.069, respectively (Ferreira et al., 2020); and from Mexico (∼0.05 for both SNPs) (Bonifaz-Peña et al., 2014; Gonzalez-Covarrubias et al., 2019). To our knowledge, the MAFs of rs1142345 and rs1800460 in the Paiter-Suruí cohort are the highest reported worldwide: the Genome Aggregation Database (https://gnomad.broadinstitute.org/) shows global MAFs of 0.011–0.055 for rs1142345 and <0.001–0.042 for rs1800460, while the corresponding values for 1KGP superpopulations are 0.017–0.067 and zero - 0.040, respectively (https://www.ensembl.org/index.html). In both databases, Asians, especially East Asians have the lowest MAF of rs1142345 and rs1800460. The large discrepancy in MAF of these SNPs between Paiter-Suruí and East Asians is unexpected, considering that there is almost consensus that the earliest Amerindians probably entered the American continent from Asia through the Bering Strait (Salzano and Bortolini, 2002). Genetic drift, which prevails among Amerindian populations (Cavalli-Sforza et al., 1994; Salzano and Bortolini, 2002), may be invoked to account for the discrepancy in MAF of *TMPT* rs1142345 and rs1800460 between East Asians and our Paiter-Suruí cohort. Genetic drift is also a likely explanation for the variable frequency of these *TPMT* and the *NUDT15* rs116855232 SNPs in the study cohorts. Allelic fluctuations of *NUDT15* rs116855232 and polymorphisms in other pharmacogenes, notably *CYP2E1*, *GSTT1*, *VKORC1*, *CYP2C9*, *CYP2C19* and *CYP2D6* across distinct Native American peoples have been previously reported, and attributed to evolutionary processes, particularly genetic drift (Gaspar et al., 2002; Perini et al., 2008; Suarez-Kurtz et al., 2019; Rodrigues et al., 2020).

The nearly perfect LD (D´ = 0.96; R2 = 0.86) between *TPMT* rs1142345 and rs1800460 SNPs in the overall study cohort is consistent with previous data for Native groups from Mexico (Bonifaz-Peña et al., 2014; Gonzalez-Covarrubias et al., 2019) and for individuals of the 1KGP_Admixed American (AMR) superpopulation with >85% Native ancestry (Suarez-Kurtz et al., 2020). For comparison, the R2 values for LD of rs1142345 and rs1800460 equals 0.69 in the overall AMR, and ranges from 0.04 to 0.96 in other 1KGP superpopulations.

The differential distribution of the genotype-inferred metabolic phenotypes across the study cohorts has important PGx implications regarding intolerance to thiopurine drugs. Accordingly, on the basis of the compound NUDT15/TPMT phenotypes associated with intolerance to thiopurines, the CPIC guideline recommendations to reduce the thiopurine initial dose or to consider a dose reduction apply to 4.4% Yanomami and 5.6% Munduruku, *versus* 41% Paiter-Suruí. The proportion of Paiter-Suruí at risk of thiopurine intolerance seems to be three- to 4-fold higher than other populations worldwide, including non-indigenous Brazilian ALL patients (Suarez-Kurtz et al., 2023). However, a nearly identical proportion (41.1%) of individuals at risk for thiopurine intolerance was reported for the above mentioned subcohort of the 1KGP_AMR superpopulation, comprising persons with >85% Native ancestry (Suarez-Kurtz et al., 2020). This subcohort differed from Paiter-Suruí in MAF of the *NUDT15* and *TPMT* risk SNPs: *TPMT* rs1142345 and rs1800460 were 3-fold more common, while *NUDT15* rs116855232 was 2-fold less common in Paiter Suruí, compared to the 1KGP subcohort.

Limitations of this study include: First, *TPMT* and *NUDT15* genotyping was restricted to the most common PGx variants listed in the CPIC guideline for thiopurines and the reference allele (*1) for each gene was assigned by default. Second, the cohorts investigated do not fully represent the diversity and heterogeneity of Native American peoples; indeed, a recent Brazilian Census identified 305 distinct Amerindian ethnicities, speaking 274 languages (https://educa.ibge.gov. br/jovens/conheca-o-brasil/populacao/20506-indigenas. html). Thus, it is necessary to evaluate the genetic profile of different groups of Brazilian natives, who are underrepresented in genetics databases and in health studies. However, this study provides relevant clinical information from three distinct native populations of the Brazilian Amazon. Third, the possibility of admixture with Brazilian non-indigenous, as a consequence of contact with people of European and, to a lesser extent, African ancestry cannot be discarded.

In conclusion, this is the first report of the distribution of clinically relevant *NUDT15* and *TPMT* SNPs and inferred compound NUDT15/TMPT metabolic phenotypes in three major Native populations from indigenous reservation areas in the Brazilian Amazon, namely Munduruku, Paiter-Suruí and Yanomami. The MAF of the interrogated SNPs, especially *TPMT* rs1142345 and rs1800460, differed significantly and largely across cohorts, consistent with the ample genetic diversity of Native American peoples. From a PGx perspective, a major finding is the large (41%), seemingly the largest reported worldwide, proportion of individuals at risk for thiopurine intolerance (41%) among Paiter-Suruí.

## Data Availability

The original contributions presented in the study are publicly available. This data can be found here: https://figshare.com/articles/dataset/Supplementary_Table_S2_-_Article_of_Indegenous_/25104014.
