# Supplementary material for: NUDT15 and TPMT polymorphisms in three distinct native populations of the Brazilian Amazon
Source: Front Pharmacol. 2024 Feb 6;15:1359570. doi: 10.3389/fphar.2024.1359570 (PMC10876798; doi:10.3389/fphar.2024.1359570)
Supplement: Supplementary file 1 [file DataSheet2.PDF]

Legend to Supplementary Figure

Supplementary Figure S1. Indigenous reservation areas in the Brazilian Amazon. The study cohorts comprised individuals from (■) Munduruku (Sawré Muybu Indigenous land), (▲) Paiter-Suruí (Sete de Setembro Indigenous land) and (●) Ninam (Yanomami Indigenous land) groups.

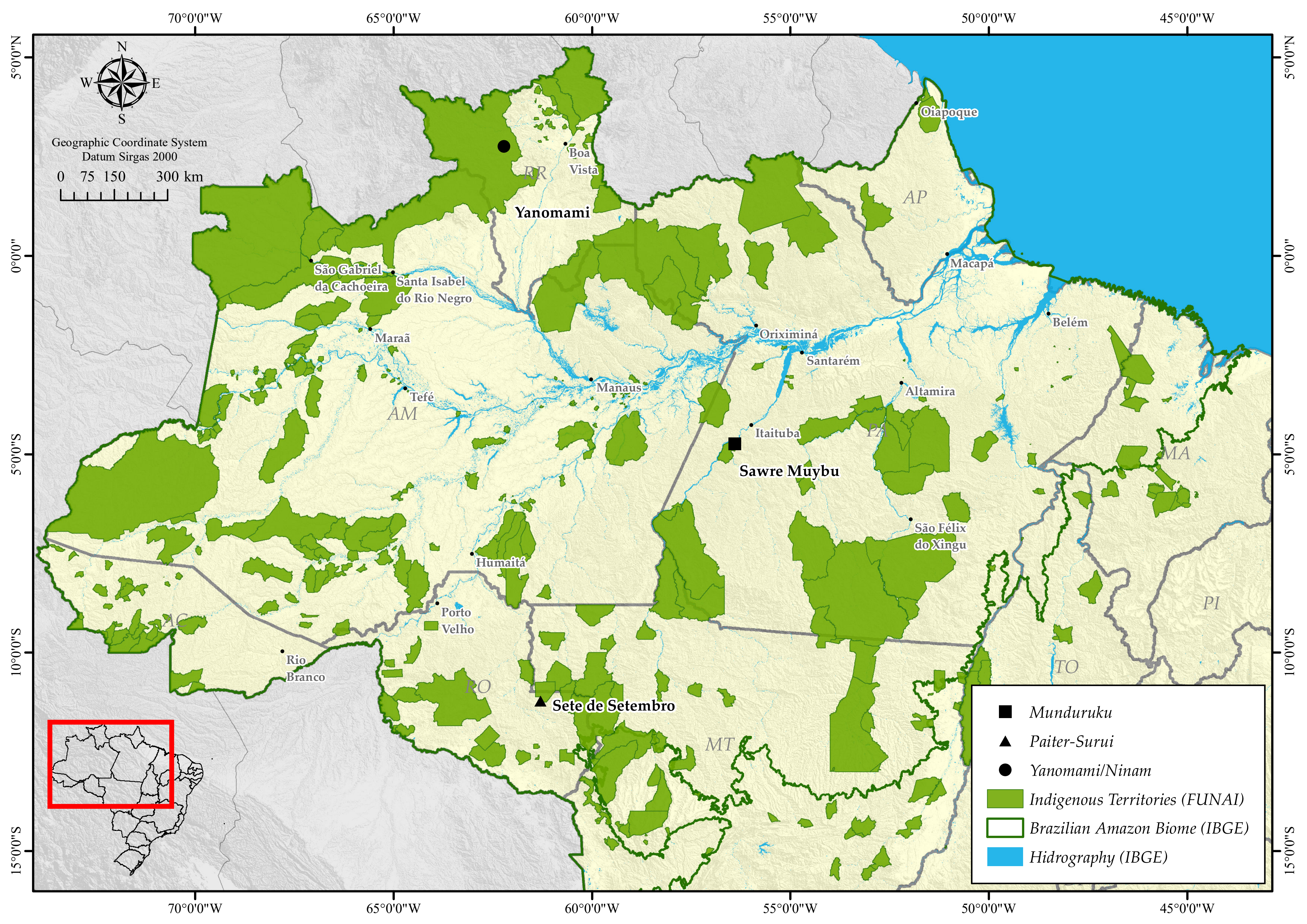
70°0'0"W
65°0'0"W
60°0'0"W
55°0'0"W
50°0'0"W
45°0'0"W
5°0'0"N
0°0'0"
5°0'0"S
10°0'0"S
15°0'0"S
N
W
E
S
Geographic Coordinate System
Datum Sirgas 2000
0 75 150 300 km
Yanomami
RR
Boa Vista
Oiapoque
AP
Macapá
São Gabriel da Cachoeira
Santa Isabel do Rio Negro
Maraã
Tefé
AM
Manaus
Oriximiná
Santarém
Belém
Altamira
Itaituba
PA
Sawre Muybu
MA
São Félix do Xingu
Humaitá
Porto Velho
AC
Rio Branco
RO
Sete de Setembro
MT
TO
PI
Munduruku
Paiter-Surui
Yanomami/Ninam
Indigenous Territories (FUNAI)
Brazilian Amazon Biome (IBGE)
Hidrography (IBGE)
