# Supplementary material for: NUDT15 and TPMT polymorphisms in three distinct native populations of the Brazilian Amazon
Source: Front Pharmacol. 2024 Feb 6;15:1359570. doi: 10.3389/fphar.2024.1359570 (PMC10876798; doi:10.3389/fphar.2024.1359570)
Supplement: Supplementary file 2 [file Table1.DOCX]

| **Supplementary Table S1.** Distribution of NUDT15, TMPT and compound metabolic phenotypes. | | | | | | |
| --- | --- | --- | --- | --- | --- | --- |
| **Enzyme** | **Phenotypes** | **YANOMAMI** | **MUNDURUKU** | **PAITER SURUÍ** |  | **Statistical Analysis** |
| NUDT15 | NM | 0.967 | 0.957 | 0.878 |  | *P* = 0.12 |
|  | IM | 0.033 | 0.033 | 0.100 |  |  |
|  | PM | 0.0 | 0.011 | 0.022 |  |  |
|  |  |  |  |  |  |  |
| TPMT | NM | 0.989 | 0.967 | 0.640 |  | *P* < 0.00001 |
|  | IM | 0.011 | 0.033 | 0.326 |  | V = 0.33 |
|  | PM | 0.0 | 0.0 | 0.034 |  |  |
|  |  |  |  |  |  |  |
| COMPOUND* | NM | 0.956 | 0.924 | 0.589 |  | *P* < 0.00001 |
|  | IM | 0.044 | 0.065 | 0.311 |  | V = 0.37 |
|  | PM | 0.0 | 0.011 | 0.056 |  |  |
|  | Compound IM | 0.0 | 0.0 | 0.044 |  |  |

NM = normal metabolizer; IM = intermediate metabolizer; PM = poor metabolizer.

* Combined metabolic phenotype of NUDT15 and TPMT: NM, normal metabolizer for both enzymes; IM = intermediate metabolizer for either enzyme; PM, poor metabolizer for either or both enzymes; Compound IM, intermediate metabolizer for both enzymes. *P* value from Chi-square test. Cramér´s V test was applied to assess the strength of statistically significant associations.
