# Supplementary material for: NUDT15 and TPMT polymorphisms in three distinct native populations of the Brazilian Amazon
Source: Front Pharmacol. 2024 Feb 6;15:1359570. doi: 10.3389/fphar.2024.1359570 (PMC10876798; doi:10.3389/fphar.2024.1359570)
Supplement: Supplementary file 3 [file DataSheet1.docx]

**Supplementary File 1**

This work is part of a fieldwork investigation carried out by a multidisciplinary and specialized team, which recruited all indigenous people who agreed to participate in the study during the period that the team was present in their respective villages in three different moments. At that moment our team carried out a kind of census in the investigated villages. In the Munduruku indigenous group, who live in Sawré Muybu Indigenous land, the fieldwork investigation was carried out between 29 October and 9 November 2019; and 112 individuals (over 12 years old) had DNA to genetic polymorphism analysis. In the Paiter-Suruí indigenous group who live in Sete de Setembro Indigenous land, the fieldwork investigation was carried out between 30 April and 5 May 2023; and 104 individuals (over 12 years) had DNA to genetic polymorphism analysis. In the Yanomami indigenous Land, from the Ninam subgroup, the fieldwork investigation was carried out between 4 and 14 October 2022; and 154 individuals (over 12 years old) had DNA to genetic polymorphism analysis.

The first records about the contact of the indigenous Munduruku people with the colonizing fronts date from the second half of the 18th century, with the first written reference being from 1768 (https://pib.socioambiental.org/pt/Povo:Munduruku#Hist.C3. B3ria_do_contato; accessed on 11/20/2023). The Munduruku are speaking of Tupi mother language and originally located in different regions and territories in the states of Pará (southwest, channel and tributaries of the Tapajós river, in the municipalities of Santarém, Itaituba, Jacareacanga), Amazonas (east, Canumã river, municipality of Nova Olinda; and close to Transamazônica, municipality of Borba), and Mato Grosso (North, Rio dos Peixes region, municipality and Juara). Today, the Munduruku population numbers around 14,000 people and is mainly concentrated in the Indigenous Land of the same name, with most of the villages located on the Cururu River, a tributary of the Tapajós. This study was conducted in the Sawré Muybu Indigenous Land. Its area is extended to 178.173 hectares and was identified by the Munduruku people themselves in 2008. Since then, the land status has not been regularized, that is, the territory has not yet been approved by the Federal Government. In the region, there are 10 villages where a population of approximately 1,000 people lives. The Munduruku population included in our study lives in the villages Sawré Muybu, Sawré Aboy and Poxo Muybu (Map), and samples were collected from October to November 2019 (Basta et al., 2021; Silva et al., 2023).

The Yanomami indigenous people constitute a society of hunter-farmers from the North of the Amazon belonging to a cultural and linguistic set composed of at least five subgroups of languages from the same family: Yanomam, Yanomamɨ, Sanöma, Ninam and Ỹaroamë. The Yanomami ancestral territory extends over approximately 23 million hectares, between Venezuela and Brazil, in the interfluve region of the Orinoco and Amazon rivers, respectively. Until the end of the 19th century, the Yanomami only maintained contact with other neighboring indigenous groups. In Brazil, the first contacts with representatives of the Limits and Borders Commission and employees of the Indian Protection Service (SPI) occurred between 1910 and 1940. From the 1940s until the mid-1960s, with the opening of some SPI posts, and several religious missions, the first points of permanent contact were established in its territory (https://pib.socioambiental.org/pt/Povo:Yanomami#Primeiros_contatos; accessed on 11/20/2023). Currently, the Yanomami number approximately 29,000 inhabitants distributed in 365 villages, occupying an area of 9,664,975 hectares of continuous forest, known as the Yanomami Indigenous Land (YIL). YIL is recognized as having traditional occupation and was demarcated and approved by the Brazilian Government on May 25, 1992. This study included Yanomami from the Ninam subgroup who live in the villages Milikowaxi, Lasasi, Ilihimakoko, Pewau, Polapi, Uxiú, Thoripi, Caju and Castanha, located on the banks of the Mucajaí River. Samples were collected in October 2022.

The Paiter-Suruí people speak the Tupi-Mondé language. They currently live on the border of the states of Rondônia and Mato Grosso, in the northern region of the country, in an area that cover 247.870 hectares, known as Sete de Setembro Indigenous Land. There are records that the Paiter-Suruí indigenous people migrated from the Mato Grosso region to Rondônia, in the 19th century, fleeing persecution from colonizers. From the end of the 19th century until the 1920s, with the exploitation of rubber, the construction of the Madeira-Mamoré railway and the installation of telegraph lines by the Rondon Commission, the migratory flow to Rondônia was great and its effects were felt on the indigenous population in the region, causing many fights and deaths. From 1940 to 1950, a new rubber economic cycle and cassiterite mining promoted significant growth in the population of the then Guaporé territory (created in 1943 and named "Território de Rondônia" in 1956). As a result of the national integration plan, during the military dictatorship in Brazil, the Cuiabá-Porto Velho highway (BR-364) was completed in 1968, and Rondônia became one of the areas with the greatest agricultural expansion in the country. Population growth from the 1950s onwards, associated with economic expansion projects, resulted in land conflicts between original peoples, farmers, rubber tappers and other extractives, forcing the Paiter-Suruí to abandon their villages again.

The Paiter-Suruí were officially contacted by the National Indian Foundation (Funai) in 1969, in the camp called Sete de Setembro. Consequently, the main village Suruí-Paiter, adjacent to the post, became known as Sete de Setembro. However, the Paiter-Suruí only began to live permanently in this area in 1973, when they came to seek medical assistance due to a measles epidemic that killed around 300 people (https://pib.socioambiental.org/pt/Povo:Surui_Paiter#Hist.C3.B3rico_do_contato; accessed on 11/20/2023). Currently, the Paiter-Surui population numbers approximately 1,500 people living in 22 villages distributed across the Sete de Setembro Indigenous Land. In this study were enrolled Paiter-Suruí people from Lapetanha, Gamir, Riozinho, Pabekepi, Joaquim, and Tikã indigenous villages and samples were collected in May 2023.
